# Supplementary material for: Measuring the dispersion of rainfall using Bayesian confidence intervals for coefficient of variation of delta-lognormal distribution: a study from Thailand
Source: PeerJ. 2019 Jul 22;7:e7344. doi: 10.7717/peerj.7344 (PMC6657683; doi:10.7717/peerj.7344)
Supplement: Supplemental Information 1 [file peerj-07-7344-s001.docx]

**Dataset S1.** Rainfall data (mm.) in July 2015 for national parks in Nan province, Thailand

| Date | NP1 | NP2 | NP3 | NP4 | NP5 | NP6 | NP7 |
| --- | --- | --- | --- | --- | --- | --- | --- |
| 1 | 0 | 0 | 0 | 0 | 0 | 0 | 0 |
| 2 | 0 | 0 | 0 | 0 | 0 | 0 | 0 |
| 3 | 0 | 0 | 0 | 0 | 0 | 0 | 0 |
| 4 | 0 | 0 | 0 | 0 | 0 | 0 | 0 |
| 5 | 1.9 | 0 | 0 | 0 | 0 | 0 | 0 |
| 6 | 47.9 | 0 | 0 | 0 | 0 | 0 | 0 |
| 7 | 10.5 | 0 | 8.9 | 2.55 | 12.0 | 0 | 0 |
| 8 | 43.8 | 3.0 | 3.7 | 39.5 | 46.0 | 8.6 | 4.5 |
| 9 | 1.9 | 0 | 0.7 | 0 | 0 | 10.8 | 0 |
| 10 | 0.6 | 0 | 10.5 | 0 | 0 | 0 | 0 |
| 11 | 5.6 | 0 | 28.2 | 5.0 | 21.0 | 63.9 | 0 |
| 12 | 0 | 0 | 25.7 | 27.5 | 0 | 0 | 0 |
| 13 | 3.1 | 0 | 13.4 | 17.5 | 0 | 30.0 | 0 |
| 14 | 3.2 | 0 | 0 | 0 | 0 | 9.4 | 0 |
| 15 | 13.8 | 0 | 14.6 | 5.0 | 0 | 18.6 | 0 |
| 16 | 3.0 | 0 | 7.0 | 17.5 | 6.0 | 8.6 | 10.0 |
| 17 | 7.4 | 0 | 36.2 | 0 | 0 | 4.7 | 0 |
| 18 | 14.5 | 0 | 3.2 | 3.75 | 0 | 2.5 | 0 |
| 19 | 34.2 | 13.0 | 40.0 | 25.0 | 12.0 | 10.0 | 0 |
| 20 | 14.9 | 0 | 16.7 | 17.5 | 0 | 4.0 | 10.0 |
| 21 | 15.8 | 36.0 | 54.2 | 30.0 | 73.0 | 35.3 | 30.0 |
| 22 | 3.8 | 0 | 4.8 | 2.5 | 7.0 | 6.3 | 0 |
| 23 | 42.1 | 0 | 19.0 | 75.0 | 13.0 | 23.1 | 10.0 |
| 24 | 23.2 | 0 | 9.1 | 16.8 | 0 | 13.9 | 0 |
| 25 | 60.7 | 6.0 | 6.7 | 53.75 | 0 | 6.7 | 0 |
| 26 | 23.7 | 10.0 | 9.8 | 37.5 | 0 | 25.5 | 0 |
| 27 | 22.5 | 0 | 32.5 | 3.75 | 0 | 10.0 | 20.0 |
| 28 | 12.4 | 0 | 12.1 | 13.75 | 10.0 | 7.2 | 0 |
| 29 | 19.6 | 18.0 | 21.3 | 0 | 0 | 8.2 | 15.0 |
| 30 | 9.2 | 10.0 | 20.0 | 7.5 | 0 | 4.0 | 35.0 |
| 31 | 13.4 | 22.0 | 19.1 | 0 | 0 | 1.3 | 35.0 |

**Note:** NP1, NP2, NP3, NP4, NP5, NP6, and NP7 represent Doi Phu Kha, Mae Charim, Nanthaburi, Tham Sa Koen, Sri Nan, Khun Sathan, and Doi Pha Klong national parks, respectively.

(http://www.parophrae.com/paro13kpi/index.php/45-2558)
