# Supplementary material for: Measuring the dispersion of rainfall using Bayesian confidence intervals for coefficient of variation of delta-lognormal distribution: a study from Thailand
Source: PeerJ. 2019 Jul 22;7:e7344. doi: 10.7717/peerj.7344 (PMC6657683; doi:10.7717/peerj.7344)
Supplement: Supplemental Information 2 [file peerj-07-7344-s002.docx]

**Dataset S2.** Rainfall data (mm.) in August 2018 from eight precipitation stations in Nan province, Thailand

| Date | S1 | S2 | S3 | S4 | S5 | S6 | S7 | S8 |
| --- | --- | --- | --- | --- | --- | --- | --- | --- |
| 1 | 1.9 | 1.0 | 0.1 | 1.3 | 0.0 | 0.0 | 0.0 | 0.0 |
| 2 | 0.0 | 8.7 | 0.0 | 0.0 | 0.0 | 0.0 | 0.0 | 0.0 |
| 3 | 0.0 | 59.0 | 3.2 | 22.6 | 81.0 | 0.0 | 0.0 | 6.4 |
| 4 | 0.0 | 0.0 | 0.0 | 0.0 | 0.0 | 0.0 | 0.0 | 0.0 |
| 5 | 0.0 | 0.7 | 0.0 | 0.0 | 5.9 | 0.0 | 0.5 | 0.0 |
| 6 | 0.0 | 0.0 | 0.0 | 2.2 | 0.0 | 0.0 | 0.4 | 0.0 |
| 7 | 0.0 | 0.0 | 5.4 | 0.0 | 0.0 | 0.0 | 1.2 | 0.0 |
| 8 | 0.7 | 0.0 | 1.6 | 1.2 | 4.6 | 1.0 | 0.0 | 2.0 |
| 9 | 9.3 | 7.1 | 19.3 | 26.3 | 8.6 | 5.2 | 22.5 | 7.0 |
| 10 | 0.0 | 0.0 | 0.0 | 0.0 | 0.8 | 0.0 | 2.8 | 0.0 |
| 11 | 0.0 | 0.0 | 4.2 | 0.0 | 0.7 | 0.0 | 1.2 | 0.0 |
| 12 | 0.0 | 2.1 | 6.0 | 0.7 | 0.0 | 0.0 | 3.7 | 1.9 |
| 13 | 0.8 | 10.6 | 9.8 | 6.4 | 12.5 | 0.0 | 8.3 | 8.4 |
| 14 | 0.4 | 1.3 | 0.4 | 2.0 | 0.0 | 0.0 | 0.0 | 12.1 |
| 15 | 0.0 | 0.0 | 0.0 | 0.0 | 0.0 | 0.0 | 0.0 | 2.7 |
| 16 | 20.7 | 126.1 | 8.0 | 15.0 | 10.4 | 370.0 | 7.3 | 2.1 |
| 17 | 69.2 | 72.0 | 106.1 | 111.0 | 151.5 | 65.0 | 90.2 | 153.0 |
| 18 | 2.1 | 3.5 | 22.5 | 0.0 | 10.5 | 3.5 | 24.4 | 3.7 |
| 19 | 0.0 | 0.0 | 0.5 | 0.0 | 0.0 | 0.0 | 0.0 | 0.0 |
| 20 | 8.9 | 2.4 | 25.6 | 2.3 | 2.3 | 0.0 | 0.0 | 31.6 |
| 21 | 8.8 | 4.4 | 4.8 | 6.7 | 5.0 | 0.0 | 2.3 | 2.0 |
| 22 | 32.8 | 9.8 | 29.6 | 3.2 | 20.6 | 2.6 | 13.0 | 36.1 |
| 23 | 1.8 | 2.6 | 9.1 | 1.4 | 0.0 | 9.0 | 2.8 | 0.0 |
| 24 | 1.1 | 4.5 | 3.6 | 2.4 | 38.4 | 14.6 | 10.0 | 4.6 |
| 25 | 8.7 | 0.0 | 1.3 | 2.0 | 8.7 | 3.5 | 0.0 | 1.6 |
| 26 | 0.0 | 0.8 | 33.8 | 8.3 | 18.5 | 0.0 | 2.7 | 14.0 |
| 27 | 6.9 | 0.1 | 5.4 | 22.4 | 31.0 | 2.2 | 7.2 | 1.3 |
| 28 | 3.3 | 0.1 | 0.7 | 2.0 | 1.0 | 0.0 | 0.7 | 0.0 |
| 29 | 2.5 | 4.8 | 6.6 | 6.0 | 3.9 | 3.7 | 12.4 | 4.7 |
| 30 | 0.0 | 1.8 | 0.5 | 0.0 | 1.8 | 1.3 | 9.2 | 0.0 |
| 31 | 0.0 | 0.3 | 3.9 | 0.0 | 6.2 | 0.0 | 20.2 | 3.3 |

**Note:** S1, S2, S3, S4, S5, S6, S7, and S8 represent Muang, Thawangpha, Thung Chang, Pua, Song Khwae, Santisuk, Chaloem Phra Kiat, and Chiang Klang precipitation stations, respectively.

(https://hydro-1.net/Data/HD-04/houly/rain_today_search.php?storage)
