# Supplementary material for: Measuring the dispersion of rainfall using Bayesian confidence intervals for coefficient of variation of delta-lognormal distribution: a study from Thailand
Source: PeerJ. 2019 Jul 22;7:e7344. doi: 10.7717/peerj.7344 (PMC6657683; doi:10.7717/peerj.7344)
Supplement: Supplemental Information 3 [file peerj-07-7344-s003.pdf]

```
##################################################################################
#      R.code for Bayesian methods (Equitailed confidence intervals and HPD) and FGCI          #
##################################################################################

library(EnvStats)
library(invgamma)
library(HDInterval)
CI.CV <- function(M=15000,m=5000,n=50,var=2,mean=0,delta=0.5,alpha=0.05){
  starttime <- proc.time()

  cv <- sqrt(exp(var)-1)                                #coefficient of variation of the LN distribution
  ex <- delta*exp(mean+var/2)                                      #mean of the delta-LN distribution
  var_x <- delta*exp((2*mean)+var)*(exp(var)-delta)         #variance of the delta-LN distribution
  eta <- sqrt(var_x)/ex                                 #coefficient of variation of the delta-LN distribution

  var.indj <- matrix(rep(0,m))
  delta.indj <- matrix(rep(0,m))
  beta.indj <- matrix(rep(0,m))
  eta.indj <- matrix(rep(0,m))

  var.jrule <- matrix(rep(0,m))
  delta.jrule <- matrix(rep(0,m))
  beta.jrule <- matrix(rep(0,m))
  eta.jrule <- matrix(rep(0,m))

  var.uni <- matrix(rep(0,m))
  delta.uni <- matrix(rep(0,m))
  beta.uni <- matrix(rep(0,m))
  eta.uni <- matrix(rep(0,m))

  L.rBaye_indj <- numeric()
  U.rBaye_indj <- numeric ()
  CIr1 <- c(0,0)
  CP.rBaye_indj <- numeric()
  Length.rBaye_indj <- numeric()

  L.rBaye_jrule <- numeric()
  U.rBaye_jrule <- numeric ()
  CIr2 <- c(0,0)
  CP.rBaye_jrule <- numeric()
  Length.rBaye_jrule <- numeric()

  L.rBaye_uni <- numeric()
```

```
U.rBaye_uni <- numeric ()
CIr3 <- c(0,0)
CP.rBaye_uni <- numeric()
Length.rBaye_uni <- numeric()

hpd_indj <- matrix(rep(0,2))
L.rBaye_hpd_indj <- numeric()
U.rBaye_hpd_indj <- numeric ()
Cir4 <- c(0,0)
CP.rBaye_hpd_indj <- numeric()
Length.rBaye_hpd_indj <- numeric()

hpd_jrule <- matrix(rep(0,2))
L.rBaye_hpd_jrule <- numeric()
U.rBaye_hpd_jrule <- numeric ()
Cir5 <- c(0,0)
CP.rBaye_hpd_jrule <- numeric()
Length.rBaye_hpd_jrule <- numeric()

hpd_uni <- matrix(rep(0,2))
L.rBaye_hpd_uni <- numeric()
U.rBaye_hpd_uni <- numeric ()
Cir6 <- c(0,0)
CP.rBaye_hpd_uni <- numeric()
Length.rBaye_hpd_uni <- numeric()

U <- matrix(rep(0,m))
beta1 <- matrix(rep(0,m))
beta2 <- matrix(rep(0,m))
var_GFQ <- matrix(rep(0,m))
delta_GFQ <- matrix(rep(0,m))
R.GFQ <- matrix(rep(0,m))

L.rGFI <- numeric()
U.rGFI <- numeric ()
CIr <- c(0,0)
CP.rGFI <- numeric()
Length.rGFI <- numeric()

i = 0
while(i < M){                                                #Begin loop i
  x <- rzmlnormAlt(n,exp(mean+var/2),cv,1-delta)     #Generate x from delta-LN distribution
  nonzero_value <- log(x[which(x!=0)])               #Find values of non-zero in population
```

```
  size_nonzero <- length(nonzero_value)                    #Find size of non-zero in population
  if(size_nonzero < n*delta){
   next
  }

  i=i+1
  print(i)

  mean_hat <- mean(nonzero_value)
  var_hat <- var(nonzero_value)
  delta_hat <- size_nonzero/n

####################################################################################
#                              Bayesian method – Equitailed confidence intervals                              #
####################################################################################

  ########################### Independent Jeffreys Prior ###########################

  beta.indj <- rbeta(m,size_nonzero+(1/2),(n-size_nonzero)+(1/2))           #Generate beta.indj
  var.indj <- rinvgamma(m, (size_nonzero-1)/2,
                ((size_nonzero-1)*var_hat)/2, scale = 1/rate)           #Compute delta-LN variance
  delta.indj <- beta.indj                                                    #Compute delta.indj
  eta.indj <- sqrt((exp(var.indj)-delta.indj)/delta.indj)                   #Compute CV of delta-LN
  L.rBaye_indj[i] <- quantile(eta.indj,alpha/2)                             #Compute lower bound
  U.rBaye_indj[i] <- quantile(eta.indj,(1-alpha/2))                         #Compute upper bound
  CIr1 <- rbind(c(L.rBaye_indj[i],U.rBaye_indj[i]))
  CP.rBaye_indj[i] <- ifelse(L.rBaye_indj[i]< eta && eta <U.rBaye_indj[i],1,0)
  Length.rBaye_indj[i] <- U.rBaye_indj[i]-L.rBaye_indj[i]           #Compute length

  ############################## Jeffreys' Rule Prior ##############################

  beta.jrule <- rbeta(m,size_nonzero+(3/2),(n-size_nonzero)+(1/2))          #Generate beta.jrule
  var.jrule <- rinvgamma(m, size_nonzero/2,
                  (size_nonzero*var_hat)/2, scale = 1/rate)             #Compute delta-LN variance
  delta.jrule <- beta.jrule                                                  #Compute delta.jrule
  eta.jrule <- sqrt((exp(var.jrule)-delta.jrule)/delta.jrule)               #Compute CV of delta-LN
  L.rBaye_jrule[i] <- quantile(eta.jrule,alpha/2)                           #Compute lower bound
  U.rBaye_jrule[i] <- quantile(eta.jrule,(1-alpha/2))                       #Compute upper bound
  CIr2 <- rbind(c(L.rBaye_jrule[i],U.rBaye_jrule[i]))
  CP.rBaye_jrule[i] <- ifelse(L.rBaye_jrule[i]< eta && eta <U.rBaye_jrule[i],1,0)
  Length.rBaye_jrule[i] <- U.rBaye_jrule[i]-L.rBaye_jrule[i]        #Compute length
```

```
################################# Uniform Prior #################################

beta.uni <- rbeta(m,size_nonzero+1,(n-size_nonzero)+1)          #Generate beta.uni
var.uni <- rinvgamma(m, (size_nonzero-2)/2,
             ((size_nonzero-2)*var_hat)/2, scale = 1/rate)      #Compute delta-LN variance
delta.uni <- beta.uni                                           #Compute delta.uni
eta.uni <- sqrt((exp(var.uni)-delta.uni)/delta.uni)             #Compute CV of delta-LN
L.rBaye_uni[i] <- quantile(eta.uni,alpha/2)                     #Compute lower bound
U.rBaye_uni[i] <- quantile(eta.uni,(1-alpha/2))                 #Compute upper bound
CIr3 <- rbind(c(L.rBaye_uni[i],U.rBaye_uni[i]))
CP.rBaye_uni[i] <- ifelse(L.rBaye_uni[i]< eta && eta <U.rBaye_uni[i],1,0)
Length.rBaye_uni[i] <- U.rBaye_uni[i]-L.rBaye_uni[i]            #Compute length


###############################################################################
#                          Bayesian method – HPD                              #
###############################################################################

########################## Independent Jeffreys Prior ##########################

hpd_indj <- hdi(eta.indj,0.95)                                  #Compute hpd interval
L.rBaye_hpd_indj[i] <- hpd_indj[1]                              #Compute lower bound
U.rBaye_hpd_indj[i] <- hpd_indj[2]                              #Compute upper bound
CIr4 <- rbind(c(L.rBaye_hpd_indj[i],U.rBaye_hpd_indj[i]))
CP.rBaye_hpd_indj[i] <- ifelse(L.rBaye_hpd_indj[i]< eta && eta <U.rBaye_hpd_indj[i],1,0)
Length.rBaye_hpd_indj[i] <- U.rBaye_hpd_indj[i]-L.rBaye_hpd_indj[i]        #Compute length

############################## Jeffreys' Rule Prior ##############################

hpd_jrule <- hdi(eta.jrule,0.95)                                #Compute hpd interval
L.rBaye_hpd_jrule[i] <- hpd_jrule[1]                            #Compute lower bound
U.rBaye_hpd_jrule[i] <- hpd_jrule[2]                            #Compute upper bound
CIr5 <- rbind(c(L.rBaye_hpd_jrule[i],U.rBaye_hpd_jrule[i]))
CP.rBaye_hpd_jrule[i] <- ifelse(L.rBaye_hpd_jrule[i]< eta && eta <U.rBaye_hpd_jrule[i],1,0)
Length.rBaye_hpd_jrule[i] <- U.rBaye_hpd_jrule[i]-L.rBaye_hpd_jrule[i]     #Compute length

################################# Uniform Prior #################################

hpd_uni <- hdi(eta.uni,0.95)                                    #Compute hpd interval
L.rBaye_hpd_uni[i] <- hpd_uni[1]                                #Compute lower bound
U.rBaye_hpd_uni[i] <- hpd_uni[2]                                #Compute upper bound
CIr6 <- rbind(c(L.rBaye_hpd_uni[i],U.rBaye_hpd_uni[i]))
CP.rBaye_hpd_uni[i] <- ifelse(L.rBaye_hpd_uni[i]< eta && eta <U.rBaye_hpd_uni[i],1,0)
```

```
   Length.rBaye_hpd_uni[i] <- U.rBaye_hpd_uni[i]-L.rBaye_hpd_uni[i]          #Compute length


################################################################################
#                                    FGCI                                      #
################################################################################

   U <- rchisq(m,(size_nonzero-1))                          #Generate U from chi-square with n(1)-1 df
   beta1 <- rbeta(m,size_nonzero,(n-size_nonzero)+1)
   beta2 <- rbeta(m,size_nonzero+1,n-size_nonzero)
   var_GFQ <- ((size_nonzero-1)*var_hat)/U             #Compute GFQ for delta-LN variance
   delta_GFQ <- (1/2)*beta1+(1/2)*beta2                #Compute GFQ for delta
   R.GFQ <-  sqrt((exp(var_GFQ)-delta_GFQ)/delta_GFQ)   #Compute GFQ for CV of delta-LN
   L.rGFI[i] <- quantile(R.GFQ,alpha/2)                       #Compute lower bound
   U.rGFI[i] <- quantile(R.GFQ,(1-alpha/2))                   #Compute upper bound
   CIr7 <- rbind(c(L.rGFI[i],U.rGFI[i]))
   CP.rGFI[i] <- ifelse(L.rGFI[i]< eta && eta <U.rGFI[i],1,0)
   Length.rGFI[i] <- U.rGFI[i]-L.rGFI[i]                      #Compute length

  }                                                           #end loop i

  ACP.rBaye_indj <- mean(CP.rBaye_indj)                       #compute CP
  Ex.length.rBaye_indj <- mean(Length.rBaye_indj)             #compute EL
  ACP.rBaye_jrule <- mean(CP.rBaye_jrule)
  Ex.length.rBaye_jrule <- mean(Length.rBaye_jrule)
  ACP.rBaye_uni <- mean(CP.rBaye_uni)
  Ex.length.rBaye_uni <- mean(Length.rBaye_uni)
  ACP.rBaye_hpd_indj <- mean(CP.rBaye_hpd_indj)
  Ex.length.rBaye_hpd_indj <- mean(Length.rBaye_hpd_indj)
  ACP.rBaye_hpd_jrule <- mean(CP.rBaye_hpd_jrule)
  Ex.length.rBaye_hpd_jrule <- mean(Length.rBaye_hpd_jrule)
  ACP.rBaye_hpd_uni <- mean(CP.rBaye_hpd_uni)
  Ex.length.rBaye_hpd_uni <- mean(Length.rBaye_hpd_uni)
  ACP.rGFI <- mean(CP.rGFI)                   #compute CP of FGCI
  Ex.length.rGFI <- mean(Length.rGFI)         #compute EL of FGCI
  elapseTime = proc.time()-starttime
  cat("M =",M,"m =",m,"n =",n,"var =",var,"delta =",delta,"\n",
     "E(x)=",n*delta,"cv=",cv,"eta=",eta,"\n",
     "CP|rBaye_indj =",ACP.rBaye_indj,"Length|rBaye_indj =",Ex.length.rBaye_indj,"\n",
     "CP|rBaye_jrule =",ACP.rBaye_jrule,"Length|rBaye_jrule =",Ex.length.rBaye_jrule,"\n",
     "CP|rBaye_uni =",ACP.rBaye_uni,"Length|rBaye_uni =",Ex.length.rBaye_uni,"\n",
     "CP|rBaye_hpd_indj =",ACP.rBaye_hpd_indj,"Length|rBaye_hpd_indj
=",Ex.length.rBaye_hpd_indj,"\n",
```

```
      "CP|rBaye_hpd_jrule =",ACP.rBaye_hpd_jrule,"Length|rBaye_hpd_jrule
=",Ex.length.rBaye_hpd_jrule,"\n",
      "CP|rBaye_hpd_uni =",ACP.rBaye_hpd_uni,"Length|rBaye_hpd_uni
=",Ex.length.rBaye_hpd_uni,"\n",
      "CP|rGFI =",ACP.rGFI,"Length|rGFI =",Ex.length.rGFI)
  result <- c("M =",M,"m =",m,"n =",n,"var =",var,"delta =",delta,"\n",
              "E(x_1)=",n*delta,"cv=",cv,"eta=",eta,"\n",
              "CP|rBaye_indj =",ACP.rBaye_indj,"Length|rBaye_indj =",Ex.length.rBaye_indj,"\n",
              "CP|rBaye_jrule =",ACP.rBaye_jrule,"Length|rBaye_jrule
=",Ex.length.rBaye_jrule,"\n",
              "CP|rBaye_uni =",ACP.rBaye_uni,"Length|rBaye_uni =",Ex.length.rBaye_uni,"\n",
              "CP|rBaye_hpd_indj =",ACP.rBaye_hpd_indj,"Length|rBaye_hpd_indj
=",Ex.length.rBaye_hpd_indj,"\n",
              "CP|rBaye_hpd_jrule =",ACP.rBaye_hpd_jrule,"Length|rBaye_hpd_jrule
=",Ex.length.rBaye_hpd_jrule,"\n",
              "CP|rBaye_hpd_uni =",ACP.rBaye_hpd_uni,"Length|rBaye_hpd_uni
=",Ex.length.rBaye_hpd_uni,"\n",
             "CP|rGFI =",ACP.rGFI,"Length|rGFI =",Ex.length.rGFI,"\n",
              "Time =",elapseTime["elapsed"])
  return(result)
}
```

```
################################################################################
#                    R.code to construct histogram of rainfall data for Example 1                        #
################################################################################

rainfall_1 <- c(0,0,0,0,1.9,47.9,10.5,43.8,1.9,0.6,5.6,0,3.1,3.2,13.8,3,7.4,14.5,34.2,14.9,15.8,3.8,
                42.1,23.2,60.7,23.7,22.5,12.4,19.6,9.2,13.4,0,0,0,0,0,0,0,3,0,0,0,0,0,0,0,0,0,0,13,0,
                36,0,0,0,6,10,0,0,18,10,22,0,0,0,0,0,0,8.9,3.7,0.7,10.5,28.2,25.7,13.4,0,14.6,7,
                36.2,3.2,40,16.7,54.2,4.8,19,9.1,6.7,9.8,32.5,12.1,21.3,20,19.1,0,0,0,0,0,0,2.55,
                39.5,0,0,5,27.5,17.5,0,5,17.5,0,3.75,25,17.5,30,2.5,75,16.8,53.75,37.5,3.75,13.75,
                0,7.5,0,0,0,0,0,0,0,12,46,0,0,21,0,0,0,0,6,0,0,12,0,73,7,13,0,0,0,0,10,0,0,0,0,0,0,0,
                0,0,0,8.6,10.8,0,63.9,0,30,9.4,18.6,8.6,4.7,2.5,10,4,35.3,6.3,23.1,13.9,6.7,25.5,10,
                7.2,8.2,4,1.3,0,0,0,0,0,0,0,4.5,0,0,0,0,0,0,0,10,0,0,0,10,30,0,10,0,0,0,20,0,15,35,
                35)

jpeg("Figure1.jpeg", width = 4, height = 4, units = 'in', res = 300)
hist(rainfall_1, ylim=c(0,140), main="The density of rainfall in July 2015", xlab="Rainfall
        (mm.)", breaks="FD", xlim=c(0,80),col="white")
dev.off()


################################################################################
#                    R.code to construct histogram of rainfall data for Example 2                        #
################################################################################

rainfall_2 <- c(1.9,0.0,0.0,0.0,0.0,0.0,0.0,0.7,9.3,0.0,0.0,0.0,0.8,0.4,0.0,20.7,69.2,2.1,0.0,8.9,8.8,
                32.8,1.8,1.1,8.7,0.0,6.9,3.3,2.5,0.0,0.0,1.0,8.7,59.0,0.0,0.7,0.0,0.0,0.0,7.1,0.0,0.0,
                2.1,10.6,1.3,0.0,126.1,72.0,3.5,0.0,2.4,4.4,9.8,2.6,4.5,0.0,0.8,0.1,0.1,4.8,1.8,0.3,
                0.1,0.0,3.2,0.0,0.0,0.0,5.4,1.6,19.3,0.0,4.2,6.0,9.8,0.4,0.0,8.0,106.1,22.5,0.5,25.6,
                4.8,29.6,9.1,3.6,1.3,33.8,5.4,0.7,6.6,0.5,3.9,1.3,0.0,22.6,0.0,0.0,2.2,0.0,1.2,26.3,
                0.0,0.0,0.7,6.4,2.0,0.0,15.0,111.0,0.0,0.0,2.3,6.7,3.2,1.4,2.4,2.0,8.3,22.4,2.0,6.0,
                0.0,0.0,0.0,0.0,81.0,0.0,5.9,0.0,0.0,4.6,8.6,0.8,0.7,0.0,12.5,0.0,0.0,10.4,151.5,10.5,
                0.0,2.3,5.0,20.6,0.0,38.4,8.7,18.5,31.0,1.0,3.9,1.8,6.2,0.0,0.0,0.0,0.0,0.0,0.0,0.0,
                1.0,5.2,0.0,0.0,0.0,0.0,0.0,0.0,370.0,65.0,3.5,0.0,0.0,0.0,2.6,9.0,14.6,3.5,0.0,2.2,
                0.0,3.7,1.3,0.0,0.0,0.0,0.0,0.0,0.5,0.4,1.2,0.0,22.5,2.8,1.2,3.7,8.3,0.0,0.0,7.3,90.2,
                24.4,0.0,0.0,2.3,13.0,2.8,10.0,0.0,2.7,7.2,0.7,12.4,9.2,20.2,0.0,0.0,6.4,0.0,0.0,0.0,
                0.0,2.0,7.0,0.0,0.0,1.9,8.4,12.1,2.7,2.1,153.0,3.7,0.0,31.6,2.0,36.1,0.0,4.6,1.6,14.0,
                1.3,0.0,4.7,0.0,3.3)

jpeg("Figure3.jpeg", width = 4, height = 4, units = 'in', res = 300)
hist(rainfall_2, ylim=c(0,140), main="The density of rainfall in August 2018", xlab="Rainfall
(mm.)", breaks="FD", xlim=c(0,80),col="white")
dev.off()
```

```
###################################################################################
#                    R.code to compute AIC values and to construct the normal Q-Q plot                    #
#                                         of rainfall data for Example 1                                   #
###################################################################################

library(MASS)
x1 <- c(1.9,47.9,10.5,43.8,1.9,0.6,5.6,3.1,3.2,13.8,3,7.4,14.5,34.2,14.9,15.8,3.8,42.1,23.2,60.7,
        23.7,22.5,12.4,19.6,9.2,13.4,3,13,36,6,10,18,10,22,8.9,3.7,0.7,10.5,28.2,25.7,13.4,14.6,
        7,36.2,3.2,40,16.7,54.2,4.8,19,9.1,6.7,9.8,32.5,12.1,21.3,20,19.1,2.55,39.5,5,27.5,17.5,5,
        17.5,3.75,25,17.5,30,2.5,75,16.8,53.75,37.5,3.75,13.75,7.5,12,46,21,6,12,73,7,13,10,8.6,
        10.8,63.9,30,9.4,18.6,8.6,4.7,2.5,10,4,35.3,6.3,23.1,13.9,6.7,25.5,10,7.2,8.2,4,1.3,4.5,10,
        10,30,10,20,15,35,35)

################################## AIC values ###################################

L.norm <- fitdistr(x1,"normal")
L.ln <- fitdistr(x1,"lognormal")
L.cau <- fitdistr(x1,"cauchy")
L.exp <- fitdistr(x1,"exponential")
AIC(L.norm)
AIC(L.ln)
AIC(L.cau)
AIC(L.exp)

################################ Normal Q-Q plot ################################

y1 <- log(x1)
jpeg("Figure2.jpeg", width = 4, height = 4, units = 'in', res = 300)
qqnorm(y1, xlab = "Theoretical Quantiles", ylab = "Sample Quantiles", plot.it = TRUE,
        datax = FALSE)
qqline(y1, datax = FALSE, distribution = qnorm, probs = c(0.25, 0.75), qtype = 7)
dev.off()
```

```
####################################################################################
#                   R.code to compute AIC values and to construct the normal Q-Q plot                  #
#                                                   of rainfall data for Example 2                                                   #
####################################################################################

library(MASS)
set.seed(100)
x2 <- c(1.9,0.7,9.3,0.8,0.4,20.7,69.2,2.1,8.9,8.8,32.8,1.8,1.1,8.7,6.9,3.3,2.5,1.0,8.7,59.0,0.7,7.1,
        2.1,10.6,1.3,126.1,72.0,3.5,2.4,4.4,9.8,2.6,4.5,0.8,0.1,0.1,4.8,1.8,0.3,0.1,3.2,5.4,1.6,19.3,
        4.2,6.0,9.8,0.4,8.0,106.1,22.5,0.5,25.6,4.8,29.6,9.1,3.6,1.3,33.8,5.4,0.7,6.6,0.5,3.9,1.3,
        22.6,2.2,1.2,26.3,0.7,6.4,2.0,15.0,111.0,2.3,6.7,3.2,1.4,2.4,2.0,8.3,22.4,2.0,6.0,81.0,5.9,
        4.6,8.6,0.8,0.7,12.5,10.4,151.5,10.5,2.3,5.0,20.6,38.4,8.7,18.5,31.0,1.0,3.9,1.8,6.2,1.0,
        5.2,370.0,65.0,3.5,2.6,9.0,14.6,3.5,2.2,3.7,1.3,0.5,0.4,1.2,22.5,2.8,1.2,3.7,8.3,7.3,90.2,
        24.4,2.3,13.0,2.8,10.0,2.7,7.2,0.7,12.4,9.2,20.2,6.4,2.0,7.0,1.9,8.4,12.1,2.7,2.1,153.0,
        3.7,31.6,2.0,36.1,4.6,1.6,14.0,1.3,4.7,3.3)

################################### AIC values ###################################

L.norm <- fitdistr(x2,"normal")
L.ln <- fitdistr(x2,"lognormal")
L.cau <- fitdistr(x2,"cauchy")
L.exp <- fitdistr(x2,"exponential")
AIC(L.norm)
AIC(L.ln)
AIC(L.cau)
AIC(L.exp)

################################# Normal Q-Q plot #################################

y2 <- log(x2)
jpeg("Figure4.jpeg", width = 4, height = 4, units = 'in', res = 300)
qqnorm(y2, xlab = "Theoretical Quantiles", ylab = "Sample Quantiles", plot.it = TRUE,
        datax = FALSE)
qqline(y2, datax = FALSE, distribution = qnorm, probs = c(0.25, 0.75), qtype = 7)
dev.off()
```
